# Expression and clinical significance of the Trop-2 gene in advanced non-small cell lung carcinoma

**DOI:** 10.3892/ol.2013.1368

**Published:** 2013-05-29

**Authors:** AIGUI JIANG, XIAOYAN GAO, DEGENG ZHANG, LIXIN ZHANG, HUIYU LU

**Affiliations:** 1 Departments of Respiratory Medicine, Taizhou People’s Hospital Affiliated to Medical College of The Nantong University, Taizhou, Jiangsu 225300, P.R. China; 2 Oncology, Taizhou People’s Hospital Affiliated to Medical College of The Nantong University, Taizhou, Jiangsu 225300, P.R. China; 3 Institute of Clinical Medicine, Taizhou People’s Hospital Affiliated to Medical College of The Nantong University, Taizhou, Jiangsu 225300, P.R. China

**Keywords:** non-small cell lung carcinoma, trop-2, prognosis, survival

## Abstract

The Trop-2 gene has been examined in various carcinomas and is reported to be significantly associated with prognosis. Little is known with regard to Trop-2 gene expression in advanced non-small cell lung carcinoma (NSCLC). The present study investigated the expression of Trop-2 and its association with the prognosis of advanced NSCLC. The clinical records of 87 patients with advanced NSCLC, consisting of 37 cases of squamous cell carcinoma (SCC) and 50 cases of adenocarcinoma (AdC), together with 17 tumor-adjacent normal tissues, were retrospectively evaluated. Trop-2 expression was measured using an immunohistochemical method and its association with clinicopathological data and prognosis was also evaluated. The expression of Trop-2 was significantly higher in the cancer tissues compared with the tumor-adjacent normal tissues, and significantly higher in SCC compared with AdC (P=0.018). In SCC, the overexpression of Trop-2 was only correlated with the histological grade of the tumor (P= 0.035) and no correlation was observed with gender, age, lymph node metastasis, TNM stage or Eastern Cooperative Oncology Group (ECOG) performance status (PS). In AdC, the over-expression of Trop-2 was correlated with the histological grade, lymph node metastasis and TNM stage (P= 0.01, 0.024 and 0.015, respectively), while no correlation with gender, age or ECOG-PS was observed. The survival frequency was significantly higher in the Trop-2-negative patients compared with the Trop-2-positive patients [17.25 months (95% CI, 14.922–19.577) vs. 13.274 months (95% CI, 11.507–15.041); P= 0.008]. The survival time was significantly longer in the Trop-2-negative AdC patients [17.275 months (95% CI, 14.575–19.975) vs. 11.469 months (95% CI, 11.507–15.041); P= 0.002], but not in the SCC patients [17.167 months (95% CI, 12.428–21.906) vs. 14.647 months (95% CI, 12.062–17.232); P= 0.276]. The multivariate analysis revealed that Trop-2 expression [hazard ratio (HR) 2.381; P= 0.038], TNM stage (HR, 2.193; P= 0.03) and ECOG-PS (HR, 2.696; P= 0.007) were independent predictors for the survival outcome of patients with AdC. These results suggest that Trop-2 overexpression is closely correlated with an unfavorable prognosis in advanced NSCLC. Trop-2 is an independent prognostic marker and a potential new therapeutic target in advanced AdC.

## Introduction

Although there has been considerable progress in recent years in the development of anti-cancer therapies, including chemotherapy, radiation therapy and biological targeted therapy, the mortality of patients with advanced non-small cell lung cancer (NSCLC) remains high due to the inability to treat the condition surgically (1). As a consequence, the research into novel prognostic biomarkers and therapeutic target structures in advanced NSCLC remains a focus of attention.

The human trophoblast cell-surface antigen, Trop-2, is a transmembrane glycoprotein originally found to be expressed at high levels on the surface of trophoblastic cells (2). More recent studies have identified Trop-2 to be highly expressed in a number of human epithelial tumors, including colorectal cancer (3), oral squamous cell carcinoma (4) and pancreatic cancer (5), and high expression is often associated with a poor prognosis. By contrast, Trop-2 expression is minimal or absent in normal epithelial tissues. This means that Trop-2 may promote tumor cell proliferation and aggressiveness (6). Trop-2 expression has also been detected in the early stage of NSCLC, but its clinical significance in operative NSCLC remains controversial (7,8). To the best of our knowledge, Trop-2 expression in advanced NSCLC and its association with prognosis has not yet been reported. In the present retrospective study, Trop-2 antigen expression and its correlation with clinicopathological features was evaluated in advanced NSCLC.

## Patients and methods

### Patients

The clinical records of 87 patients (61 males and 26 females; mean age 63.4 years; range, 45–71 years) with advanced NSCLC who were admitted to the Taizhou People’s Hospital (Taizhou, Jiangsu, China) between June 2008 and June 2010 were retrospectively evaluated. In total, 37 cases of squamous-cell carcinoma (SCC), 50 cases of adenocarcinoma (AdC) and 17 tumor-adjacent normal tissue samples were obtained. All cases were confirmed using CT-guided percutaneous or bronchoscopic lung biopsies. The patients were divided into stage IIIb and stage IV tumor groups, according to the TNM system (9). The standard procedure that was used for the inoperable stage IIIb patients was that of sequential chemo-radiation. Platinum-based doublets in two to three cycles were administered prior to irradiation. The platinum doublets were similar to those used for stage IV tumors. Radiation was administered using a linear accelerator (≥6 MeV) or cobalt-60, for a total dose of 50–60 Gy, delivered in 25–30 fractions of 2 Gy/day, 5 days/week. Patients were excluded from the study if they had received prior chemotherapy or radiotherapy, had no definitive histological diagnosis, had a bad performance status (PS; ECOG ≥3), had brain tumor metastasis or if they had a disease other than lung cancer that may have affected survival, including cardiac dysfunction, renal insufficiency, liver cirrhosis or concomitant malignancy. This study was approved by the Ethics Committee of Taizhou People’s Hospital, Jiangsu, China and was performed according to the Declaration of Helsinki. Written informed consent was obtained from each patient’s family.

### Immunohistochemistry

Paraffin-embedded tissue blocks were cut into 4-*μ*m sections and analyzed immunohistochemically (EliVision™ Plus IHC kit; Wuhan Boster Biological Engineering Co., Ltd., Wuhan, Hubei, China) for Trop-2 expression (1:50; goat polyclonal antibody; R&D Systems, Minneapolis, MN, USA). The sections were dewaxed in xylene and rehydrated using graded concentrations of ethanol. The endogenous peroxidase activity was blocked by incubating the sections in 5% hydrogen peroxide in absolute methanol at room temperature for 10 min. Antigen retrieval was performed in a microwave oven for two cycles of 10 min each. The primary antibodies were applied for 1 h at room temperature and the sections were washed three times using 0.05M Tris-buffered saline (TBS, pH 7.2), prior to 50*μ*l IgG/HRP secondary antibody (Wuhan Boster Biological Engineering Co., Ltd.) being added, followed by incubation for 30 min at room temperature. The sections were washed three times with TBS and the reaction products were visualized with diaminobenzidine (DAB kit; Wuhan Boster Biological Engineering Co., Ltd.). The sections were counterstained with hematoxylin and eosin, dehydrated and evaluated under a light microscope.

### Immunohistochemistry scoring

Positive staining for Trop-2 expression was assessed in 10 high-power fields of each tumor by two independent pathologists using light microscopy in a blinded fashion. Trop-2 expression was evaluated for each tissue sample by calculating a total immunostaining score as the product of a proportion and intensity score (5). The proportion score described the estimated fraction of positively-stained tumor cells (0, none; 1, ≤10%; 2, 10–50%; 3, 51–80%; and 4, ≥80%). The intensity score represented the estimated staining intensity (0, no staining; 1, weak; 2, moderate; and 3, strong). Thus, the total score ranged from 0–12. The positive and negative expression of Trop-2 were defined as a score of >4 and ≤4, respectively.

### Follow-up

The patients were followed up from the date of the pathological diagnosis until the date of mortality or the last follow-up at the outpatient department. At the time of the last follow-up, 80 patients (92%) had succumbed to the tumor and 7 patients (8%) were lost to follow-up or succumbed to other causes.

### Statistical analysis

The statistical analysis was performed using the SPSS 13.0 software (SPSS, Inc., Chicago, IL, USA). The associations between Trop-2 immunostaining and the clinicopathological parameters (gender, age, histologic grade, lymph node metastasis, TNM stage and ECOG-PS) were analyzed using χ^2^ and Fisher’s exact tests. Overall survival (OS) was calculated from the date of diagnosis to the date of the last follow up or mortality. The cases of patients that were lost to follow-up or had succumbed from any other cause were defined as censored data for the analysis of survival rates. The survival curves were plotted using the Kaplan-Meier method, and P-values were calculated using the log-rank test. A multivariate analysis was performed using the Cox-proportional hazards model to identify independent prognostic factors. P≤0.05 was considered to indicate a statistically significant difference.

## Results

### Trop-2 expression in tumor-adjacent normal tissues

**Figure 1.**
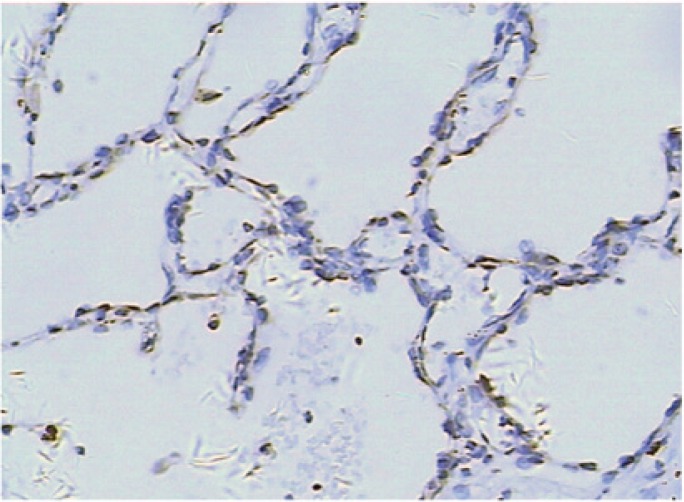
Trop-2 expression in the tumor-adjacent normal lung tissues (hematoxylin & eosin; ×400 magnification).

Trop-2 expression was absent or infrequent in the tunica mucosa bronchiorum. No positive expression was observed in the alveolar wall. Trop-2 overexpression was detected in 5.9% (1/17) of tumor adjacent normal tissues (Fig. 1).

### Trop-2 expression in advanced NSCLC

**Figure 2.**
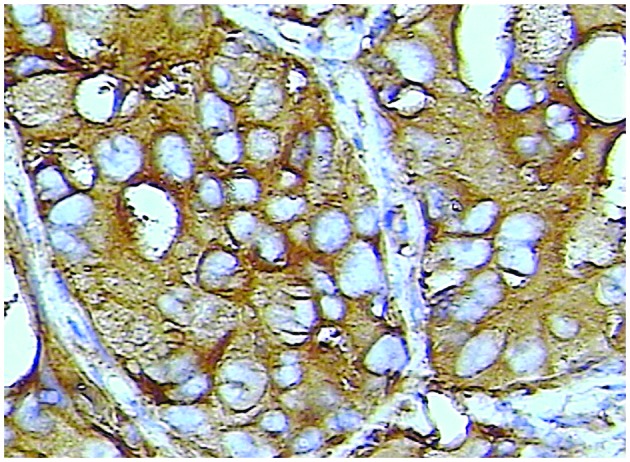
Trop-2 expression in SCC tissues (hematoxylin and eosin; ×400 magnification). SCC, squamous-cell carcinoma

**Figure 3.**
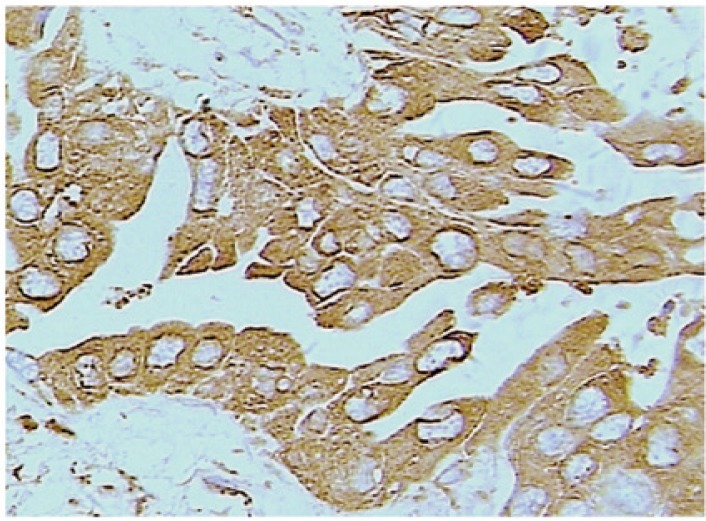
Trop-2 expression in Adc tissues (hematoxylin and eosin; ×400 magnification). AdC, adenocarcinoma.

Staining for Trop-2 occurred in a diffuse pattern localized mainly in the membrane of the cancer cells, although staining was occasionally identified in the nucleus and cytoplasm (Figs. 2 and 3). Trop-2 overexpression was detected in 52.9% (46/87) of the tumors. Trop-2 expression was higher in the advanced NSCLC tissues than in the tumor-adjacent normal tissues (P=0.000), and higher in the SCC cases [67.6% (25/37)] than in the AdC cases [42.0% (21/50); P=0.018]

### Trop-2 expression associations with clinicopathological variables and prognosis

Trop-2 overexpression in SCC did not differ significantly with regard to patient gender, age, lymph node metastasis, TNM stage or ECOG-PS. However, Trop-2 overexpression was significantly correlated with the histological grade (P=0.035; Table I). Trop-2 overexpression in AdC did not differ significantly with regard to patient gender, age or ECOG-PS. However, Trop-2 overexpression was significantly correlated with the histological grade, lymph node metastasis and TNM stage (P= 0.01, 0.024 and 0.015, respectively; Table II).

**Figure 4.**
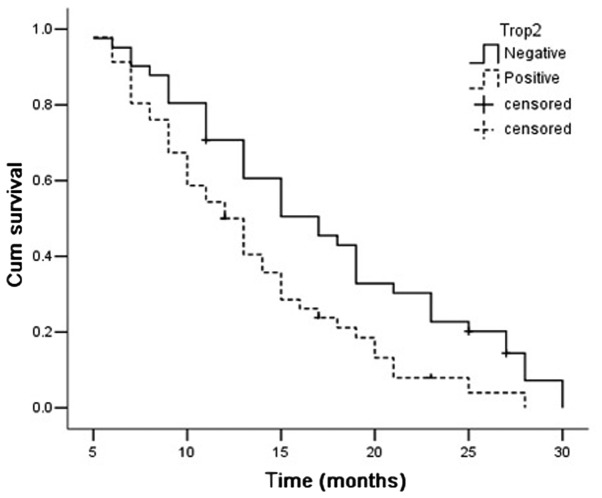
Kaplan-Meier curves in advanced NSCLC patients positive and negative for Trop-2 (Log Rank, χ^2^=7.094, P=0.008). NSCLC, non-small cell lung carcinoma; Cum, cumulative.

**Figure 5.**
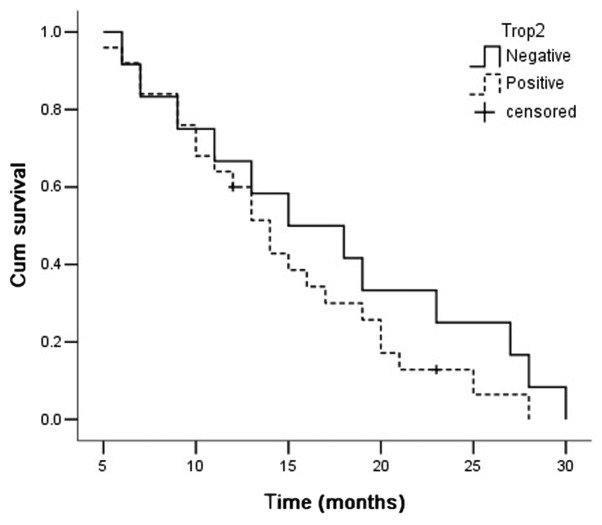
Kaplan-Meier curves in advanced squamour cell carcinoma (SCC) positive and negative for Trop-2 (Log Rank, χ^2^=1.186, P=0.276). Cum, cumulative.

**Figure 6.**
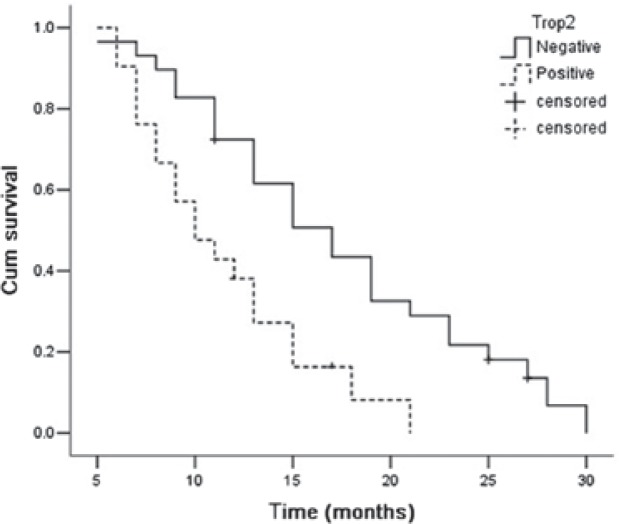
Kaplan-Meier curves in advanced adenocarcinoma (AdC) patients positive and negative for Trop-2 (Log Rank, χ^2^=9.611, P=0.002). Cum, cumulative.

The median OS time of all patients was 15.197 months (95% CI, 13.688–16.706). The survival time was significantly better in the patients with Trop-2-negative expression than those with Trop-2-positive expression [17.25 months (95% CI, 14.922–19.577) vs. 13.274 months (95% CI, 11.507–15.041); P= 0.008]. The survival time was significantly longer in the Trop-2-negative AdC patients [17.275 months (95% CI, 14.575–19.975) vs. 11.469 months (95% CI, 11.507–15.041); P=0.002], but not in the SCC patients [17.167 months (95% CI, 12.428–21.906) vs. 14.647 months (95% CI, 12.062–17.232); P=0.276; Figs. 4–6].

In the univariate survival tests of AdC that were performed using clinicopathological factors, the statistically significant parameters, other than Trop-2 expression (HR, 2.606; P= 0.004) were lymph node metastasis (HR, 2.258; P= 0.011), TNM stage (HR, 2.478; P= 0.005) and ECOG-PS (HR, 2.586; P=0.005). Other variables, including age, gender and tumor differentiation, were not associated with a better survival outcome. In the multivariate survival tests, Trop-2 expression (HR, 2.381; P= 0.038), TNM stage (HR, 2.193; P= 0.03) and ECOG-PS (HR, 2.696; P= 0.007) were identified as independent prognostic markers in advanced AdC (Table III).

## Discussion

The Trop-2 protein (also termed GA733-1, M1S1 and EGP-1), is a human trophoblast cell-surface antigen encoded by the TACSTD2 gene of human chromosome 1p32 (10). The TACSTD2 gene lacks introns and is formed by exon shuffling and retroposition of the TACSTD1 gene via an mRNA intermediate. TACSTD2 encodes a 35 kDa, type 1 transmembrane protein, which contains 323 amino acids and a single transmembrane domain. Trop-2 is a calcium channel protein that is associated with the regulation of intracellular calcium concentration (11). Moreover, Trop-2 plays a significant role in the regulation of tumor proliferation by increasing the stability of cyclin D1 or activating the signal pathway of ERKl-MAPK (12,13).

Early studies identified Trop-2 gene mutations to be associated with Gelatinous Drop-like Corneal Dystrophy (GDLD), a rare autosomal recessive genetic disease which leads to severe vision disorders and even blindness (14–16). More recently, studies have observed that Trop-2 is highly expressed in a number of human epithelial tumors compared with the restricted expression found in normal tissues, and that it is also associated with poor overall patient survival (3–5). These previous studies have revealed that Trop-2 may promote tumor proliferation, aggressiveness and metastasis (6). Trop-2 expression has also been detected in the early stage of NSCLC, but its clinical significance in operative NSCLC remains controversial (7,8). Kobayashi *et al* (7) revealed that 87 of 130 patients (67%) with small-sized pulmonary AdC (<2 cm diameter) were immunopositive for Trop-2 expression and therefore associated with a poor OS. The multivariate analysis showed that Trop-2 overexpression was an independent, unfavorable prognostic marker in AdC and NSCLC. Pak *et al* (8) reported that 64 of 164 patients (39%) with stage II or III NSCLC were immunopositive for Trop-2 expression. The Trop-2 expression in patients with AdC [23/100 (23%)] was significantly lower than that in the SCC patients [41/64 (64%)]. Trop-2 overexpression showed a better OS in the AdC patients. The inconsistent results between the two studies suggest that the biological role of Trop-2 may vary between early and advanced pulmonary AdC.

To the best of our knowledge, there are no studies with regard to the correlation between Trop-2 expression and advanced NSCLC in the English-language literature. The present study detected that Trop-2 expression occurred in the membrane of lung cancer cells and occasionally in the nucleus and cytoplasm. Trop-2 expression was higher in advanced NSCLC than in the tumor-adjacent normal tissues. These results revealed that the Trop-2 gene may be associated with tumorigenesis and that the progression of advanced NSCLC. Trop-2 expression in the present study was significantly higher in NSCLC than in the tumor-adjacent normal tissues. Additionally, Trop-2 was significantly overexpressed in SCC compared with AdC, as observed in a previous study (8). The present study also detected that Trop-2 overexpression in SCC was only correlated with the histological grade of the tumor. However, Trop-2 expression in AdC was correlated with the histological grade, lymph node metastasis and TNM stage. The multivariate analysis showed that Trop-2 is an independent prognostic marker in advanced pulmonary AdC and that it may play a more significant role in the pathogenesis of this disease.

In conclusion, Trop-2 is closely correlated with unfavorable prognostic factors in advanced NSCLC. Trop-2 is also an independent prognostic marker in advanced AdC. The present results further indicate that Trop-2 may be a potential new therapeutic target for advanced AdC. However, due to the limitations inherent in retrospective analyses, the prognostic value of Trop-2 overexpression requires further validation in larger prospective studies.

## Figures and Tables

**Table I.** Trop-2 expression in association with clinicopathological factors in SCC.

Characteristics	Number	Trop-2 overexpression		Positive rate (%)	χ^2^	P-value
		No	Yes			
Gender						
Male	25	7	18	72.0	0.6911	0.406
Female	12	5	7	58.3		
Age (years)						
≥60	19	5	14	73.7	0.6668	0.414
<60	18	7	11	61.1		
Degree of differentiation						
Low-middle	16	2	14	87.5	5.1110	0.035
High	21	10	11	52.4		
Lymph node metastasis						
No	10	5	5	50.0	1.9299	0.165
Yes	27	7	20	74.1		
TNM stage						
IIIb	20	8	12	60.0	1.1376	0.319
IV	17	4	13	76.5		
PS score						
0–1	14	6	8	57.1	1.1169	0.291
2	23	6	17	73.9		

SCC, squamous-cell carcinoma; PS, performance status.

**Table II.** Trop-2 expression in association with clinicopathological factors in AdC.

Characteristics	Number	Trop-2 overexpression		Positive rate (%)	χ^2^	P-value
		No	Yes			
Gender						
Male	36	22	14	38.9	0.5109	0.475
Female	14	7	7	50.0		
Age (years)						
≥60	28	17	11	39.3	0.1925	0.661
<60	22	12	10	45.5		
Degree of differentiation						
Low-middle	25	10	15	60.0	6.6502	0.010
High	25	19	6	24.0		
Lymph node metastasis						
No	14	12	2	14.3	6.1309	0.024
Yes	36	17	19	52.8		
TNM stage						
IIIb	29	21	8	27.6	5.8888	0.015
IV	21	8	13	61.9		
PS score						
0–1	17	12	5	29.4	1.6756	0.196
2	33	17	16	48.5		

AdC, adenocarcinoma; PS, performance status.

**Table III.** Multivariate analysis of survival in advanced pulmonary adenocarcinoma.

Parameter	Regression co-efficient	Standard error	Wald	HR (95% CI)	P-value
Trop-2 overexpression	0.868	0.417	4.325	2.381 (1.051–5.394)	0.038
Age (≥60 vs. <60)	−0.154	0.433	0.126	0.857 (0.367–2.004)	0.722
Gender (male vs. female)	0.285	0.410	0.484	1.330 (0.595–2.972)	0.487
TNM stage (IIIb vs. IV)	0.785	0.361	4.727	2.193 (1.080–4.453)	0.030
Degree of differentiation (Low-middle vs. high)	−0.362	0.429	0.714	0.696 (0.300–1.614)	0.398
Lymph node metastasis (Yes vs. no)	0.477	0.388	1.510	1.611 (0.753–3.448)	0.219
PS score (0–1 vs. 2)	0.992	0.368	7.254	2.696 (1.310–5.549)	0.007

CI, confidence interval; PS, performance status; HR, hazard ratio.
